# Supplementary material for: Sex differences in aggression and its neural substrate in a cichlid fish
Source: Sci Rep. 2025 Jan 2;15:84. doi: 10.1038/s41598-024-84188-4 (PMC11696305; doi:10.1038/s41598-024-84188-4)
Supplement: Supplementary file 8 — Supplementary Material 8 [file 41598_2024_84188_MOESM8_ESM.pdf]

Sex differences in aggression and its neural substrates in a cichlid fish

Supplementary Methods and Results

pS6 Antibody Validation

To validate the specific binding of the pS6 antibody, we performed two immunohistochemistry runs in two series of the same tissue according to the protocol described previously. In one series, the protocol was followed exactly as written, and in the other series, the primary antibody was omitted. These runs were performed in parallel with the same reagents. Stained slides were imaged according to protocol described previously and representative images are shown in Figure S8. Through this validation, we show that there is no non-specific binding of the pS6 antibody in the *A. burtoni* tissue. A preadsorption assay to test specificity of the primary antibody was not conducted.

Statistical results of comparisons between NC and mirror fish for behaviors

There was a significant effect of condition but not sex or an interaction on lateral display type 1 (Table S1; lateral display type 1, Two-way ANOVA, condition: $p = 0.1856$). There was a significant effect of sex, condition, and sex*condition on lateral display type 2 (Table S1; lateral display type 2, Two-way ANOVA, sex: $p = 0.0480$, condition: $p = 0.0137$, interaction: $p = 0.0480$). Post-hoc Šídák's tests reveal that males in the mirror condition perform more type 1 and type 2 lateral displays than negative control males, but females do not differ by condition (Table S1; lateral display type 1: $p = 0.0112$, lateral display type 2: $p = 0.0092$, Šídák's tests). Furthermore, there was a significant effect of condition, but not sex or an interaction on s-curve displays, mouth contact, and taps (Table S1; s-curve displays, Two-way ANOVA, condition: $p = 0.0072$, Table S1; mouth contact, Two-way ANOVA of log transformation, condition: $p < 0.0001$, Table S1; taps, Two-way ANOVA of log transformation, condition: $p = 0.0031$). Mirror males perform more s-curve displays than negative control males, but females do not differ by condition (Table S1; s-curve displays, Šídák's test, Male Mirror – NC, $p = 0.0078$). Both mirror males and females perform more mouth contact behaviors than negative controls (Table S1; mouth contact, Male Mirror – NC: $p < 0.0001$, Female Mirror – NC: $p < 0.0001$, Šídák's test). Mirror females perform more taps than negative control females while males do not differ by condition (Table S1; taps, Šídák's test, $p = 0.0198$).

There was a significant effect of condition but not sex or an interaction on total number of behaviors and total number of aggressive behaviors (Table S1; # of behaviors, Two-way ANOVA of log transformation, condition: $p < 0.0001$, Table S1; total aggression, Two-way ANOVA, condition: $p < 0.0001$). Both mirror males and mirror females perform more total behaviors and aggressive behaviors than negative controls (Table S1; # of behaviors, Male Mirror – NC: $p < 0.0001$, Female Mirror – NC: $p < 0.0001$; total aggression, Male Mirror – NC: $p = 0.0050$, Female Mirror – NC: $p = 0.0033$, Šídák's tests).

1

	Sex		Condition		Sex*Condition	
	F	P	F	P	F	P
Lateral Display Type 1	1.849<br>1, 26	0.1856	10.49<br>1, 26	**0.0033**	1.849<br>1, 26	0.1856
Lateral Display Type 2	4.307<br>1, 26	**0.0480**	6.995<br>1, 26	**0.0137**	4.307<br>1, 26	**0.0480**

S-curve Displays	3.637<br>1, 26	0.0676	8.501<br>1, 26	**0.0072**	3.637<br>1, 26	0.0676
Mouth Contact	0.6808<br>1, 26	0.4168	67.36<br>1, 26	**< 0.0001**	0.2122<br>1, 26	0.6489
Taps	0.1947<br>1, 26	0.6627	10.63<br>1, 26	**0.0031**	0.1218<br>1, 26	0.7299
# of Behaviors	0.4261<br>1, 26	0.5197	88.75<br>1, 26	**< 0.0001**	0.01433<br>1, 26	0.9056
Total Aggression	0.03252<br>1, 26	0.8583	23.23<br>1, 26	**< 0.0001**	0.07211<br>1, 26	0.7904

2

	Male Mirror – NC	Female Mirror – NC
	P	P
Lateral Display Type 1	**0.0112**	0.2935
Lateral Display Type 2	**0.0092**	0.8873
S-curve Displays	**0.0078**	0.6908
Mouth Contact	**< 0.0001**	**< 0.0001**
Taps	0.1293	**0.0198**
# of Behaviors	< 0.0001	< 0.0001
Total Aggression	0.0050	0.0033

Table S1. (1) Effect of sex and condition on behaviors quantified in a mirror assay. (2) Post-hoc comparisons.

	Sex		Condition		Sex*Condition	
	F	P	F	P	F	P
POA	0.8266<br>1, 25	0.3719	2.494<br>1, 25	0.1268	0.003165<br>1, 25	0.9556
nPPa	2.210<br>1, 25	0.1497	2.744<br>1, 25	0.1101	0.01917<br>1, 25	0.8910
nPMp	0.6952<br>1,19	0.4148	0.4276<br>1, 19	0.5210	0.3621<br>1, 19	0.5545
Dl-g	2.497<br>1, 24	0.1271	0.03271<br>1, 24	0.8580	1.146<br>1, 24	0.2951
Dm-3	0.7580<br>1, 25	0.3922	0.4440<br>1, 25	0.5113	3.073<br>1, 25	0.0919
Xm	2.314<br>1, 23	0.1418	2.800<br>1, 23	0.1078	0.003933<br>1, 23	0.9505

Table S2. Effect of sex and condition on pS6 immunoreactivity.

		Vs	ATn	PAG	CG	nPPa	nMMp	nPMp	POA	VL	Xm	Dm-3	Dl-g
Vv	R	-0.039	-0.115	0.262	0.156	0.494	0.332	0.394	0.431	0.36	0.499	-0.167	0.468
	P	0.881	0.66	0.309	0.551	**0.044**	0.192	0.118	0.084	0.156	**0.042**	0.523	0.058
Vs	R		-0.097	-0.214	0.573	0.371	0.393	0.478	0.365	0.101	-0.282	-0.351	0.248
	P		0.711	0.41	**0.016**	0.143	0.119	0.052	0.149	0.699	0.272	0.167	0.337
ATn	R			-0.05	-0.158	0.076	0.535	0.119	0.314	0.155	0.079	-0.061	-0.152
	P			0.848	0.544	0.771	**0.027**	0.649	0.22	0.552	0.763	0.815	0.561
PAG	R				0.387	0.119	0.001	-0.038	0.02	0.078	0.021	0.399	-0.053
	P				0.124	0.65	0.997	0.886	0.938	0.766	0.937	0.112	0.841
CG	R					0.408	0.262	0.408	0.336	0.131	-0.256	-0.09	0.267
	P					0.104	0.309	0.104	0.188	0.616	0.322	0.73	0.301
nPPa	R						0.739	0.721	0.91	-0.195	0.074	-0.411	0.097
	P						**0.001**	**0.001**	**4.12E-07**	0.454	0.779	0.101	0.712
nMMp	R							0.769	0.918	-0.134	0.265	-0.582	-0.044
	P							**3.08E-04**	**2.11E-07**	0.607	0.304	**0.014**	0.867
nPMp	R								0.845	-0.276	0.299	-0.488	-0.014
	P								**1.97E-05**	0.283	0.244	**0.047**	0.957
POA	R									-0.234	0.207	-0.527	0.049
	P									0.367	0.424	**0.03**	0.852
VL	R										-0.008	0.342	0.488
	P										0.975	0.179	**0.047**
Xm	R											-0.349	-0.224
	P											0.169	0.386
Dm-3	R												0.009
	P												0.973

Table S3. Pearson correlation values of pS6 immunoreactivity in mirror fish.

		Vs	ATn	PAG	CG	nPPa	nMMp	nPMp	POA	VL	Xm	Dm-3	Dl-g
Vv	R	0.825	0.918	0.32	0.245	0.498	0.389	0.731	0.534	0.303	0.274	0.806	0.639
	P	**0.0005**	**0.00001**	0.287	0.419	0.083	0.189	**0.005**	0.06	0.314	0.365	**0.0009**	**0.019**
Vs	R		0.789	0.216	0.471	0.636	0.443	0.821	0.66	0.583	0.653	0.903	0.703
	P		**0.00136**	0.479	0.104	**0.02**	0.129	**0.001**	**0.014**	**0.036**	**0.016**	**2.39E-05**	**0.007**
ATn	R			0.376	0.349	0.588	0.397	0.712	0.577	0.251	0.356	0.696	0.583
	P			0.206	0.242	**0.035**	0.179	**0.006**	**0.039**	0.409	0.232	**0.0083**	**0.037**
PAG	R				0.32	0.456	0.546	0.466	0.519	0.093	0.213	0.168	0.614
	P				0.287	0.117	0.054	0.108	0.069	0.764	0.486	0.5833	**0.026**
CG	R					0.412	0.245	0.456	0.462	0.682	0.658	0.279	0.346
	P					0.162	0.419	0.117	0.112	**0.01**	**0.014**	0.3552	0.247
nPPa	R						0.492	0.686	0.833	0.539	0.712	0.685	0.578
	P						0.088	**0.01**	**4.07E-04**	0.057	**0.006**	**0.0098**	**0.038**
nMMp	R							0.785	0.868	0.363	0.353	0.375	0.443
	P							**0.001**	**1.21E-04**	0.223	0.237	0.2073	0.13
nPMp	R								0.902	0.557	0.519	0.793	0.579
	P								**2.48E-05**	**0.048**	0.069	**0.0012**	**0.038**
POA	R									0.595	0.625	0.649	0.549
	P									**0.032**	**0.022**	**0.0163**	0.052
VL	R										0.865	0.613	0.504
	P										**1.32E-04**	**0.0258**	0.079
Xm	R											0.587	0.622
	P											**0.035**	**0.023**
Dm-3	R												0.653
	P												**0.016**

Table S4. Pearson correlation values of pS6 immunoreactivity in negative control fish.

	PC1	PC2
Vv	**0.30709407**	-0.1550194
Vs	**0.33475244**	-0.138937
ATn	0.0786963	0.2546581
PAG	0.25017048	-0.1925946
CG	**0.30707561**	-0.1857131
nPPa	**0.35933606**	0.1545626
nMMp	**0.34608376**	**0.32122405**
nPMp	**0.33826091**	0.29469053
POA	**0.37019927**	**0.31311545**
VL	0.25130994	**-0.3761193**
Xm	0.05941277	0.20019019
Dm-3	0.08322134	**-0.4540014**
Dl-g	0.237873	**-0.3472971**

Table S5. PCA component loadings for combined mirror and negative control fish.

	PC1	PC2
Vv	0.22776301	0.26646841
Vs	0.23783291	0.24196108
ATn	0.10442256	-0.2176296
PAG	0.00044817	0.24869525
CG	0.20889062	**0.42034068**
nPPa	**0.40851882**	0.06593241
nMMp	**0.42541373**	-0.1225907
nPMp	**0.41868272**	-0.046673
POA	**0.44725236**	-0.0550992
VL	-0.0798084	**0.43327604**
Xm	0.12684742	-0.2566584
Dm-3	-0.2984527	0.2373274
Dl-g	0.04787439	**0.50003537**

Table S6. PCA component loadings for mirror fish.

	PC1	PC2
Vv	0.2776275	**-0.3914845**
Vs	0.322159	-0.068534
ATn	0.2796261	**-0.3441852**
PAG	0.1820699	-0.1765652
CG	0.2067307	**0.42871937**

nPPa	0.2962492	0.07486987
nMMp	0.2415449	-0.1042493
nPMp	**0.3299453**	-0.1190802
POA	**0.3188154**	0.02304585
VL	0.251112	**0.49804965**
Xm	0.2684649	**0.46637798**
Dm-3	**0.306559**	-0.090484
Dl-g	0.2816647	-0.0605815

Table S7. PCA component loadings for negative control fish.

Supplementary Figures

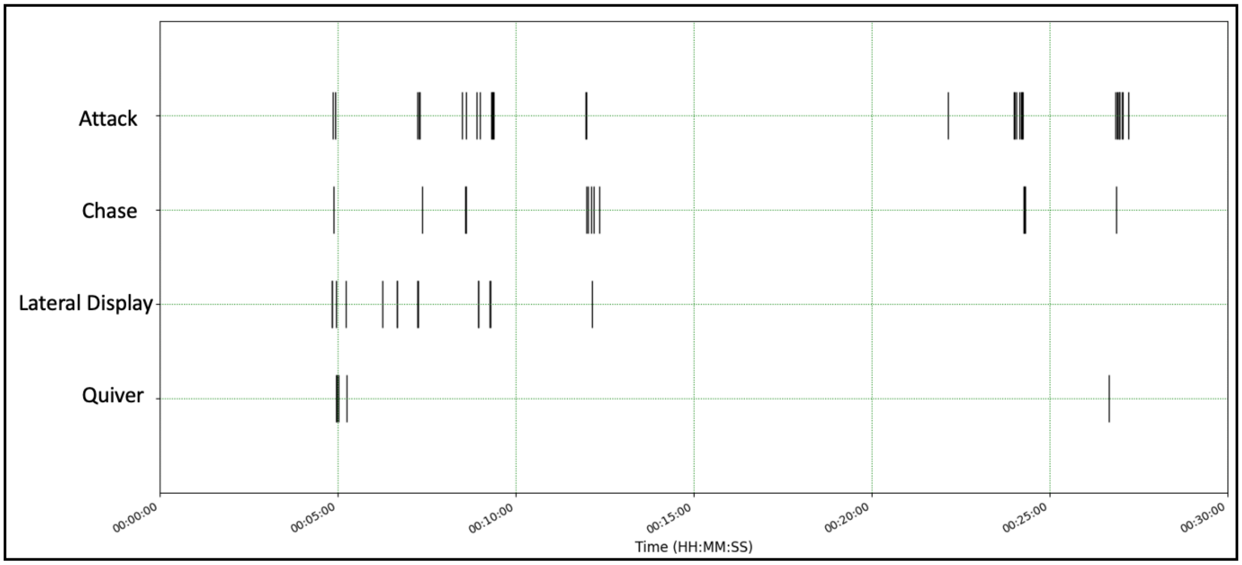

Figure S1. Representative raster plot of aggressive behaviors performed by a focal female in a dyad assay. Gravid females perform aggressive behaviors (including quivers) in an assay with a live opponent. The first 30 minutes of the behaviors performed in a dyad assay are represented here.

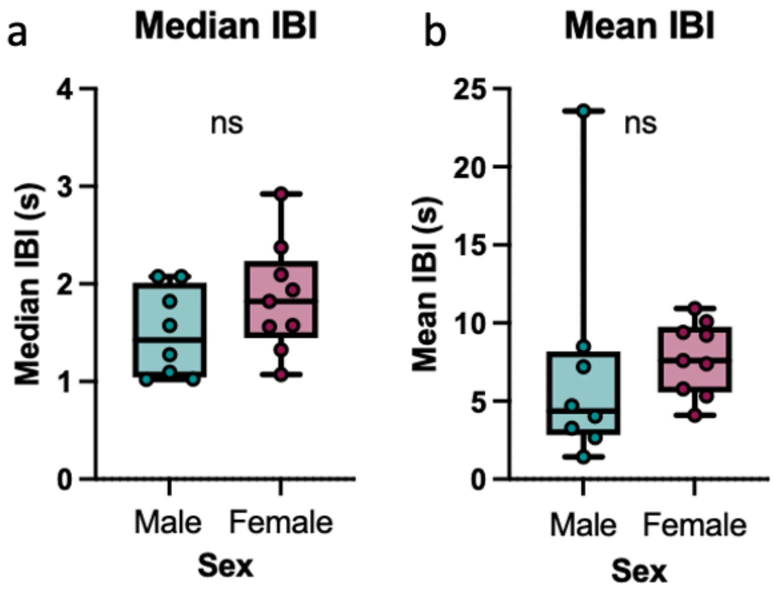

Figure S2. No sex differences in inter-behavioral intervals. (a)(b) Mirror males and females do not differ by median and mean inter-behavioral intervals.

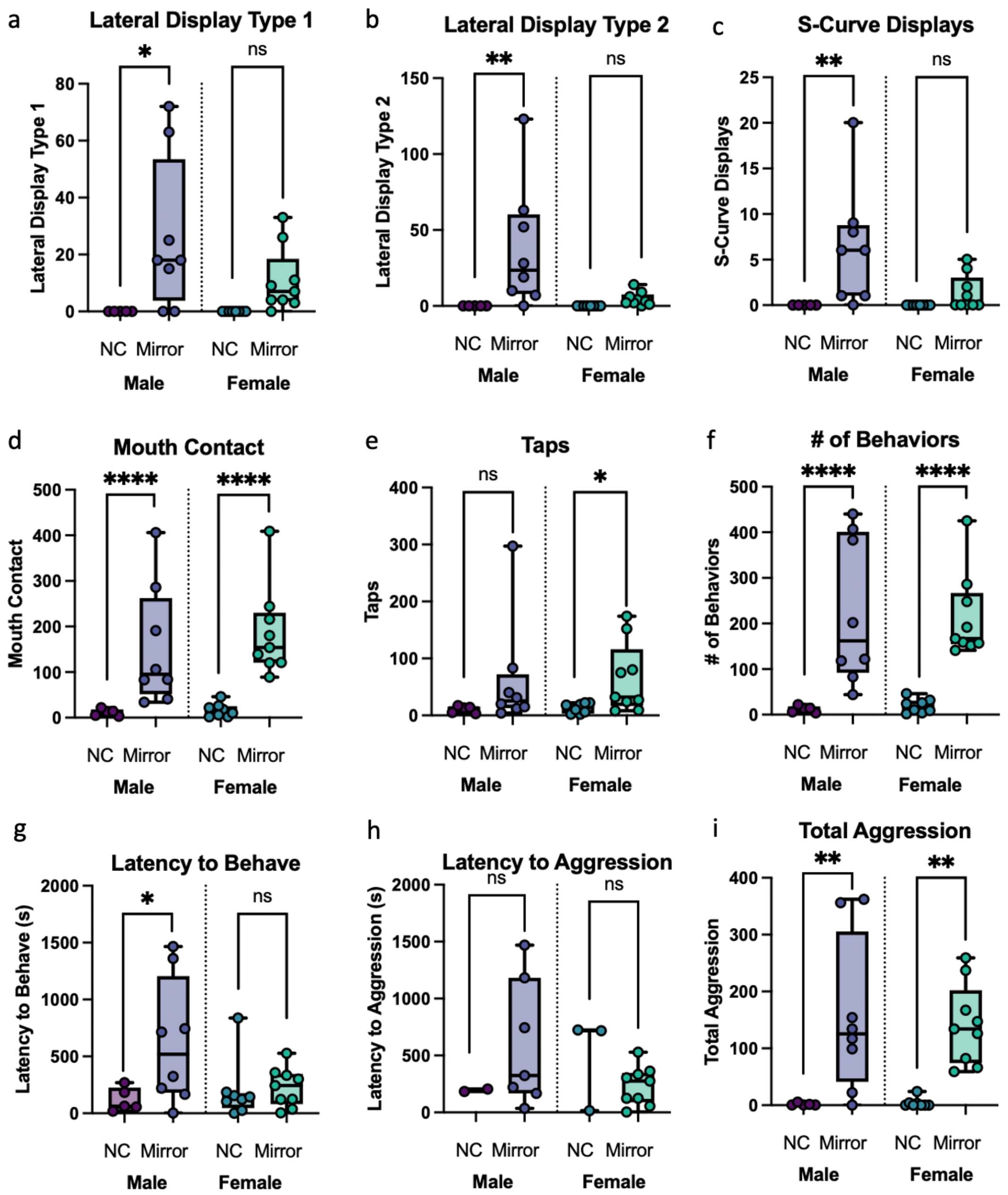

Figure S3. *A. burtoni* perform more behaviors when exposed to a mirror compared to an opaque cover. (a)(b) Mirror males perform more lateral display type 1 and 2 than negative control males while females do not differ by condition. (c) Mirror males perform more s-curve displays than negative control males while females do not differ by condition. (d) Mirror males and females perform more mouth contact behaviors than negative control fish. (e) Mirror females perform more taps than negative control females while males do not differ by condition. (f) Mirror males and females perform more behaviors than negative control fish. (g) Mirror males have a longer latency to behave than negative control males while females do not differ by condition. (h) The latency to aggression does not statistically differ by sex or condition. (i) Mirror males and females perform more aggressive behaviors than negative control fish.

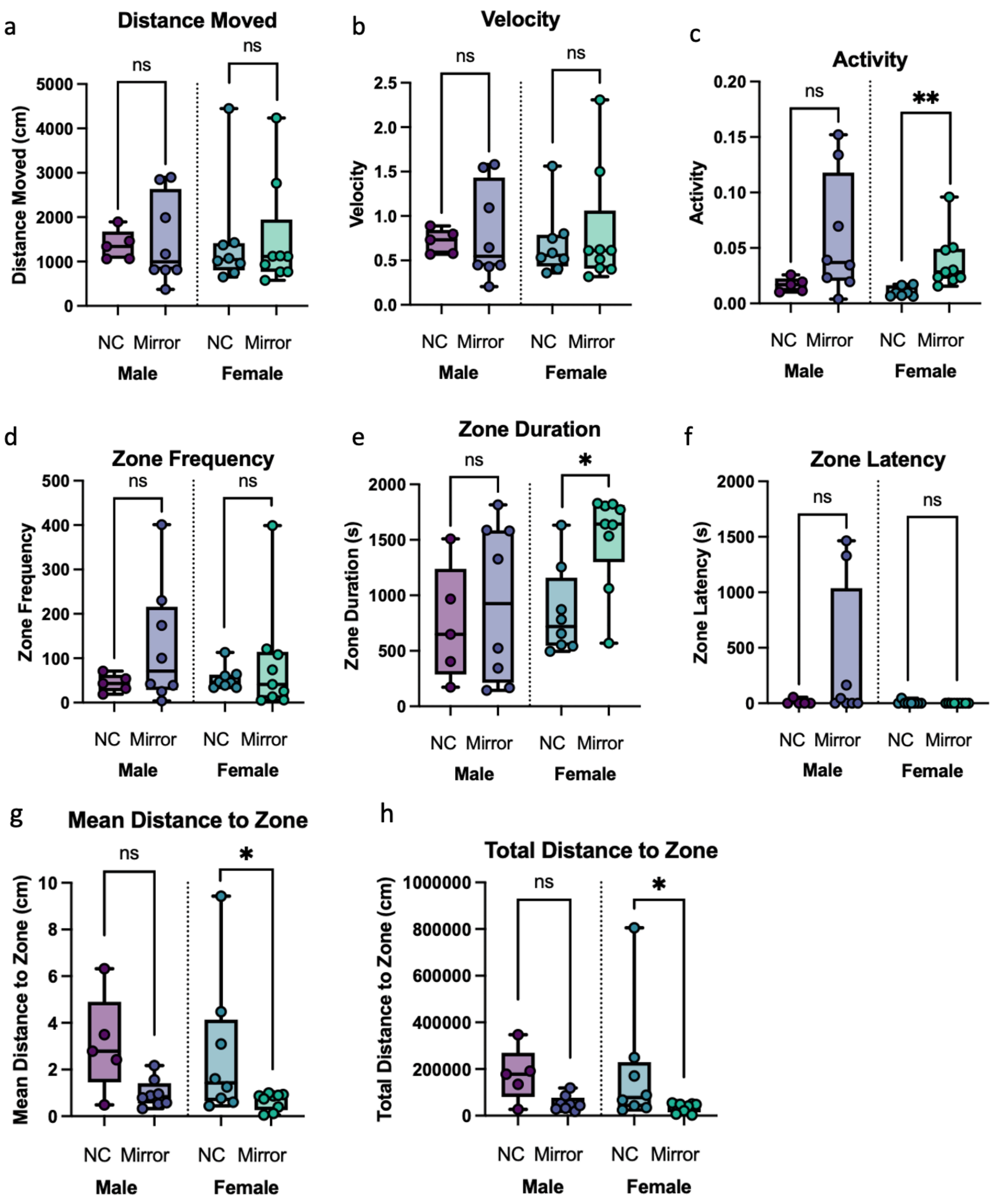

Figure S4. Similarities and differences in Noldus tracking data in fish exposed to a mirror or opaque cover. (a)(b) Distance moved and velocity did not differ by sex or condition. (c) Mirror females were more active than negative control females. Mirror males followed this pattern although it was not statistically significant. (d) Zone frequency did not differ by sex or condition. (e) Mirror females spent more time in the zone than negative control females while males did not differ by condition. (f) Zone latency did not differ by sex or condition. (g)(h) Negative control females maintained a larger mean and total distance from the zone than negative control females. Negative control males followed this pattern although it was not statistically significant.

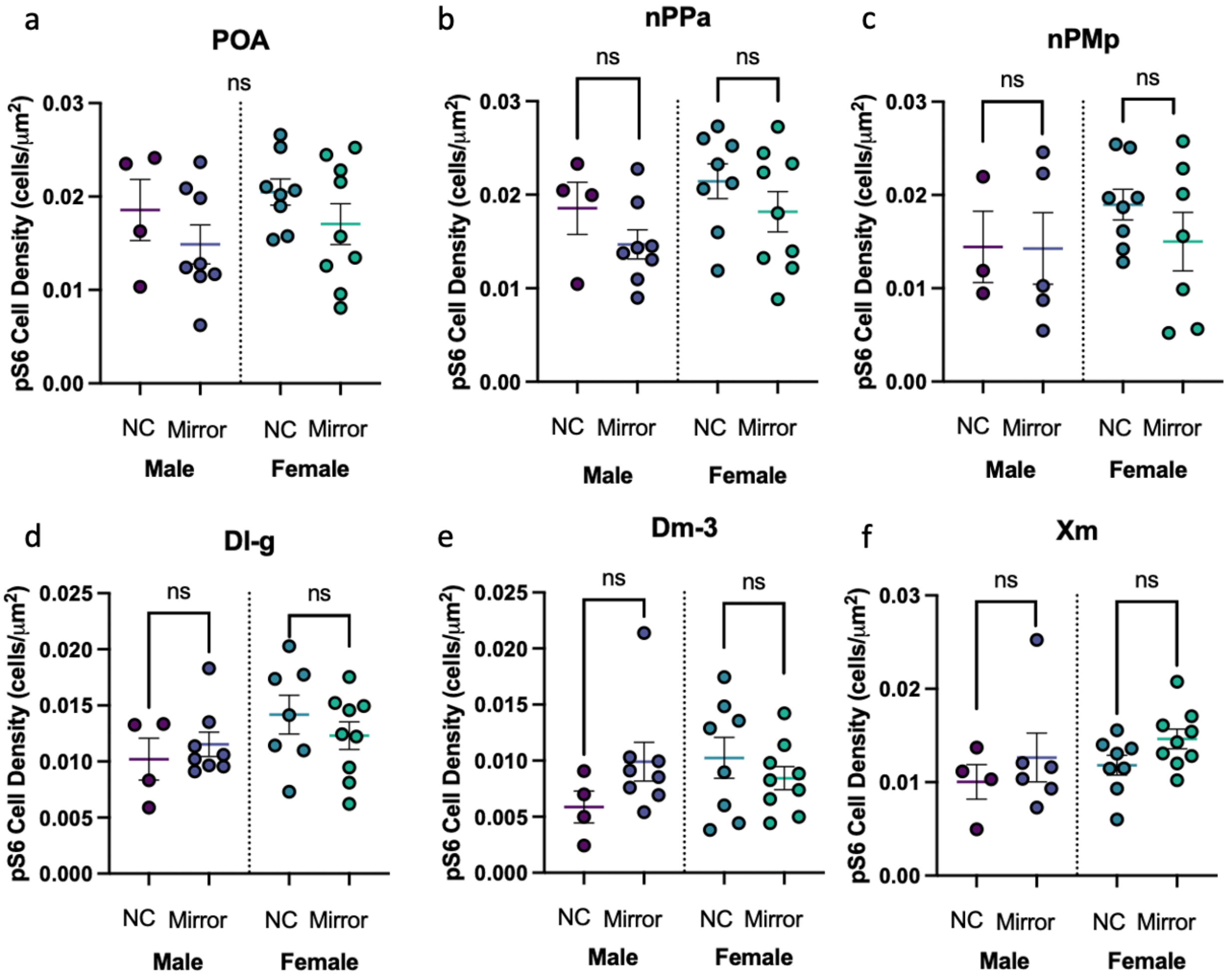

Figure S5. pS6 immunoreactivity in brain regions relevant to social behaviors and sensory processing. (a) pS6 cell density does not differ by condition or sex in the POA. (b)(c)(d)(e)(f) pS6 immunoreactivity did not differ by sex or condition in the nPPa, nPMp, Dl-g, Dm-3, or Xm.

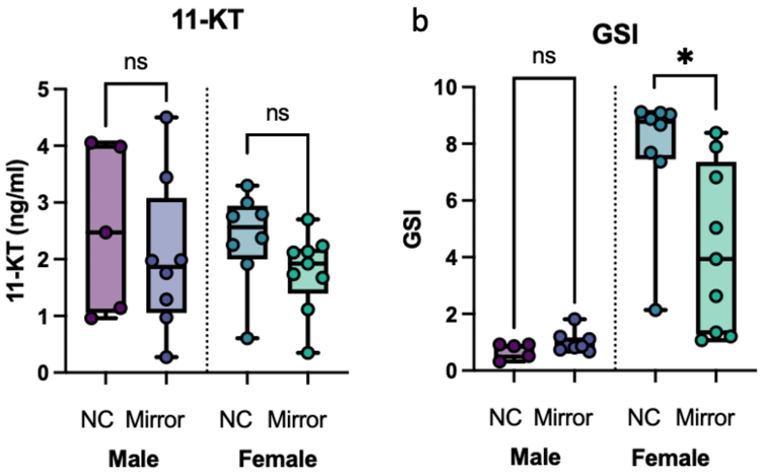

Figure S6. Physiological measures between sex and condition. (a) 11-KT did not differ between sex and condition. (b) Negative control females had a higher GSI than mirror females.

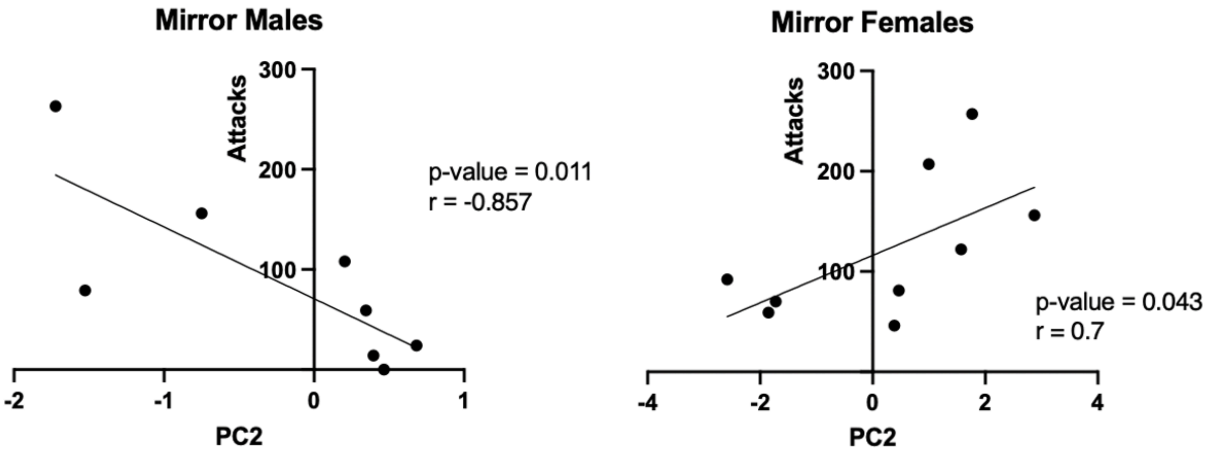

Figure S7. Correlations of PC2 to attacks for mirror males and mirror females.

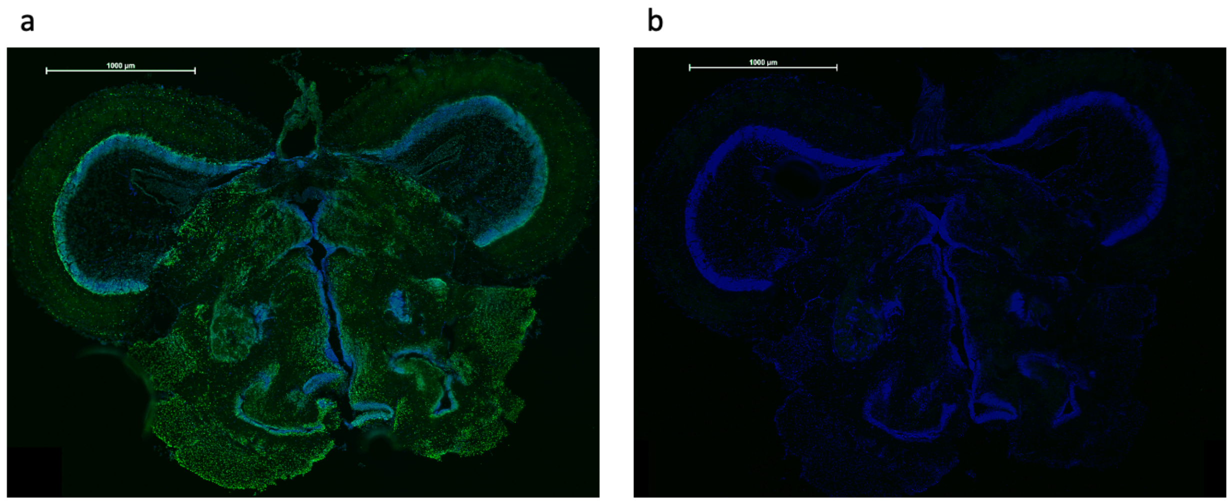

Figure S8. Validation of the pS6 antibody indicating no non-specific binding in control tissue. (a) Tissue stained according to immunohistochemistry protocol. (b) Tissue stained without primary pS6 antibody. pS6 staining is green and DAPI staining is blue. Scale bar = 1000 μm.

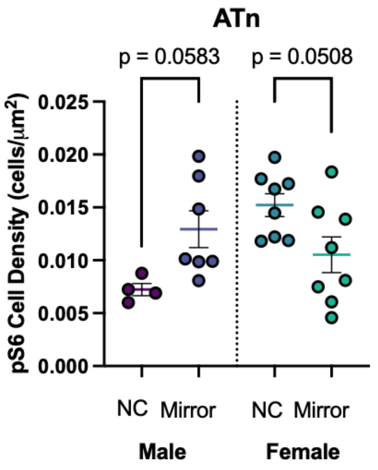

Figure S9. pS6 cell density in the ATn with mirror male outlier excluded.
